# Antihypertensive Indigenous Lebanese Plants: Ethnopharmacology and a Clinical Trial

**DOI:** 10.3390/biom9070292

**Published:** 2019-07-20

**Authors:** Ali A. Samaha, Mirna Fawaz, Ali Salami, Safaa Baydoun, Ali H. Eid

**Affiliations:** 1 Lebanese International University, Beirut, P.O. Box 146404, Lebanon; 2 Faculty of Health Sciences, Beirut Arab University, Beirut, P.O. Box 11-5020, Lebanon; 3 Lebanese University, Faculty of Public Health IV, Zahle, P.O. Box 6573/14, Lebanon; 4 Rayak University Hospital, Rayak, P.O. Box 1200, Lebanon; 5 Lebanese University, Rammal Hassan Rammal Research Laboratory, Physio-toxicity (PhyTox) Research Group, Faculty of Sciences (V), Nabatieh, P.O. Box 6573/14, Lebanon; 6 Research Center for Environment and Development, Beirut Arab University, Bekaa, P.O. Box 11-5020, Lebanon; 7 Department of Pharmacology and Toxicology, American University of Beirut, Beirut, P.O. Box 11-0236, Lebanon; 8 Department of Biomedical Sciences, Qatar University, Doha, P.O. Box 2713, Qatar

**Keywords:** hypertension, herbal medicine, *Urtica dioica*, *Viola odorata*, *Mentha longifolia*

## Abstract

Hypertension is highly prevalent among the Lebanese adult population and is indeed the major cause of mortality in Lebanon. Traditional use of antihypertensive medicinal plants has long been practiced. The aim of this study is to document this traditional knowledge and clinically test the antihypertensive capacity of three of the most commonly used wild plant species *Mentha longifolia*, *Viola odorata* and *Urtica dioica*. Ethno-pharmacological data was collected by personal interviews with herbalists and traditional healers using a semi structured survey questionnaire and assessing relative frequency of citation (RFC). The clinical study was conducted by a randomized, blind, placebo-controlled trial in 29 subjects with mild hypertension distributed in four groups, three plant extract treatments and one placebo. Systolic (SBP) and diastolic blood pressures (DBP) as well as mean arterial blood pressures (MAP) were monitored at weeks 4, 8, 12 and 16 during the treatment with 300 mL/day of plant extract. Results showed that *M. longifolia*, *U. dioica* and *V. odorata* exhibited the highest values of RCF (0.95) followed by *Allium ampeloprasum* (0.94), *Apium graveolens* (0.92) and *Crataegus azarolus* (0.90). The clinical trial revealed dose- and duration-dependent significant reductions in SBP, DBP and MAP of subjects treated with *M. longifolia*, *U. dioica* or *V. odorata*. Our findings indicate that extracts of these plants present an effective, safe and promising potential as a phyto-therapuetical approach for the treatment of mild hypertension. More research on the phytochemistry, pharmacological effects and the underlying mechanisms is necessary.

## 1. Introduction

Hypertension (HTN), commonly known as high blood pressure, remains a major contributor to morbidity and mortality associated with cardiovascular diseases (CVD) and other conditions including stroke, kidney failure, dementia, premature death and disability [1]. Recent data from 154 countries confirms an increase in hypertensive cases from 17,307 per 100,000 in 1990 to 20,525 per 100,000 in 2015 [2,3,4]. Accumulating evidence also argues that high systolic blood pressure (SBP) (≥140 mm Hg) is responsible for 143 million disabilities as well as 14% of total deaths, mostly due to CVD [2,3,4]. This is further confirmed by a meta-analysis of 1670 studies in 71 countries together involving 29.5 million participants [5]. This study indeed reveals that the prevalence of HTN ranges from 4% to 78%, with the highest worldwide blood pressure prevalence shifting from high income countries to low income countries [5]. 

In Lebanon, several studies have emphasized the extent of the burden of HTN with a prevalence of 36.9% [6], 29.3% [7] or 31.2% [8] among adults. In these studies, the control rate is reported at only 27%, 9.5% and 28.7%, respectively [6,7,8]. Alarmingly, 47% of proportional mortality in Lebanon is directly related to CVD [9]. This is not surprising given the high prevalence of CVD risk factors among the Lebanese population [10,11], among which hypertension is the most prominent [12]. 

There are many factors involved in regulating blood pressure (BP). These include cardiac output, circulatory blood volume, vascular caliber, elasticity and reactivity, hormonal mediators as well as neural stimulation. The factors that affect cardiac output include sodium intake, renal function, and mineralocorticoids. Inotropic effects occur via extracellular fluid volume augmentation as well as an increase in heart rate and contractility. As for the peripheral vascular resistance, the sympathetic nervous system (SNS), humoral factors and local autoregulation are key players [13]. SNS elicits its impact primarily by inducing vasoconstriction and promoting sodium retention. Humoral mediators include vasoconstrictors such as endothelin, angiotensin II, catecholamines or vasodilators such as nitric oxide (NO), prostaglandins and kinins [14]. Moreover, in arterial smooth muscle cells, secondary messengers, such as cyclic AMP, are well-known to modulate cellular phenotypes such as adhesion and actin cytoskeleton reorganization. Both phenotypic changes play a role in vasoconstriction and thus in peripheral vascular disease including hypertension [15,16,17,18,19,20]. In addition, blood viscosity, blood flow velocity and vascular wall conditions contribute to the regulation of BP [21].

Several drugs, belonging to different classes, are employed for the management or treatment of HTN. The main drugs available are thiazide diuretics, angiotensin-converting enzyme (ACE) inhibitors, angiotensin receptor II blockers and calcium channel blockers [22]. Additional medications that are sometimes used are vasodilators, aldosterone antagonists, β-blockers, α-blockers, renin inhibitors and central-acting agents [23]. Pharmacologic lowering of HTN in patients with disparate antihypertensive mechanisms reduces the risk of, but does not entirely prevent, HTN-related CVD events, such as stroke, heart failure, retinopathy and nephropathy [24]. Certainly, some of the remaining risk in treated cases is attributable to BP levels that remain significantly above those in normotensive individuals. 

Control of BP requires multiple antihypertensive agents in the majority of patients with hypertension [25]. The availability of multiple antihypertensive agents affords the practitioner the ability to use highly effective drug combinations that both reduce BP and protect target organs [26]. This is especially important in view of the high global prevalence of resistant hypertension [27]. Data from 3.2 million patients indicate that the prevalence of true-resistant hypertension was 22.9%, 56.0% and 12.3% in chronic kidney disease, renal transplant and elderly patients, respectively [27]. This study further confirmed the high need for new treatments for resistant hypertension [27]. 

In this context, medicinal plants with cardio-vasculoprotective, hypotensive or antihypertensive therapeutic values have been subject to enormous concerted research efforts during the last three decades [28,29,30,31,32,33]. Clinical and preclinical studies demonstrate the beneficial effects of many plants and further underscore their potential as a source for pharmaceutical drugs [15,28,29,30,31,32,34]. Interestingly, plant-derived alkaloids like reserpine, rescinnamine and serpentine are still used to treat severe forms of hypertension, with reserpine being roughly as effective as other first-line antihypertensive drugs [35]. 

In their attempts to control hypertension and its attendant complications amid the scarce socioeconomic resources, rural communities in developing countries including Lebanon and the Levant have resorted to herbal remedies. However, much scientific efforts are needed to validate the effectiveness and elucidate the safety profile of such herbal remedies [28,36]. Numerous and chemically diverse secondary metabolites that are actually optimized for exerting biological functions are still far from being exhaustively investigated. While natural product-based drug discovery and development represents a complex endeavor demanding a highly integrated interdisciplinary approach, published scientific evidence, technologic advances and research trends clearly indicate that natural products will be among the most important sources of new drugs also in the future [37,38]. Moreover, there is a clear demonstration that the rich flora biodiversity and associated ethno-pharmacological traditional knowledge of the East Mediterranean region has indeed provided humanity with many important drugs [39]. In Lebanon, despite of the citation of several native species of therapeutic value in the treatment of mild hypertension, there have been very few studies that have specifically been conducted in this regard. 

This study endeavors to document the ethno-pharmacological traditional knowledge of wild medicinal plants used in the treatment of hypertension and clinically test the blood-lowering effect of some selected species. The mechanism of action of these medicinal plants will be discussed in light of the available literature.

## 2. Materials and Methods 

This study was conducted between October 2016 and September 2018 and has been registered in the World Health Organization Clinical Trial Registry (ChiCTR1900021653) as a clinical trial. It consisted of two stages. Stage I consisted of an ethno-pharmacological survey with herbalists and traditional healers, followed by a clinical trial (stage II) to examine the effect of three of the most commonly used species. This study involved 29 subjects with pre-hypertension and two additional risk factors attending the outpatient department of Rayak University Hospital (RH), Bekaa, Lebanon but not willing to undergo any treatment by pharmaceutical medications when they will need it. Ethical approval was obtained both from Rayak Hospital and Beirut Arab University.

### 2.1. Ethnopharmacological Survery and Selection of Medicinal Plants 

Collection of traditional ethno-pharmacological knowledge comprised personal interviews with 36 herbalists and traditional healers using semi-structured questionnaires. Specimens of selected plants were collected and taxonomical identification was confirmed based on the determination keys that were described in our recent publication [40] and references therein. Quantitative data analysis was performed by computing the Relative Frequency of Citation (RFC_s_) as follows:

RFCs = FCs/N,

where *FC_s_* is frequency of citation i.e., the number of informants (herbalists and traditional healers) reporting a particular species divided by the total number of informants participating in the survey (*N*). In theory, this index varies from 0.0 to 1.0; the closer the values are to 1.0, the higher is the consensus among the informants. Three herbs of the plant species that scored a *RCF_s_* of 1.00 were selected for the clinical trial. 

### 2.2. Plant Material and Extraction Procedure

Leaves of *Mentha longifolia*, flowers of *Viola odorata* and leaves of *Urtica dioica* were collected from the wild where the species are abundantly found, namely from El Moukhtara, Jabal Moussa and Ta’anayel regions. Samples of the plant material were deposited at the Research Center for Environment and Development, Beirut Arab University. The weight of the starting material was 500 g. After air-drying, the collected material was washed with distilled water, then soaked in aqueous-methanol (30:70) for a total of three days with occasional shaking. The plant material was then filtered by a two-stage approach using muslin cloths and Whatman grade-1 filter papers (Merck, Darmstadt, Germany). This procedure was repeated twice, and the combined filtrate was condensed down to 20% of volume using rotary evaporator (Buchi, Flawil, Switzerland) at 35–40 °C under reduced pressure. A total of 5 mL of this volume was mixed with 295 mL of distilled water, which was consumed by the subjects. Placebo liquid was prepared of distilled water tinted with a food colorant to have a similar appearance to plant extracts.

### 2.3. Clinical Trial

Three medicinal plants, *M. longifolia*, *V. odorata* and *U. dioica*, were selected based on the ethnopharmacological survey and were examined for their anti-hypertensive properties on subjects with mild hypertension. The trial consisted of a 16 week, single-blind, placebo-controlled approach and was conducted under the approval of the Institutional Review Board (IRB) of Rayak Hospital (RH) that is accredited by the Ministry of Public Health, Lebanon (Approval number ECO-R-9.0-2016). The patients did not know to which group they were assigned or which herbal solution they were receiving until after the follow-up period. Block randomization was utilized to minimize bias and variability between different groups. For the purpose of obtaining this approval, a focus group consisting of a group of cardiologists and nephrologists at RH was informed of the study background, rationale and approach. Written consents assuring participating subjects that all information would be confidential and used only for research purposes were obtained. Selection criteria included subjects of 40–65 years of age with pre-hypertension and two additional risk factors such as positive family history for hypertension, sedentarity and obesity. (SBP: 135 and 139 mmHg and/or DBP: 85 and 89 mmHg) who were not willing to take any pharmaceutical medications but underwent lifestyle modifications (salt restriction) with no detectable BP lowering response. Exclusion criteria included lactating or pregnant women, history of allergy, kidney dysfunction, diabetes or any cardiovascular dysfunction. During the selection process, subjects attended two screening visits with an interval of two weeks, each of which included medical and life-style histories, physical examinations, laboratory tests and measurements of BP. In addition to general laboratory testing, blood count, creatinine and electrolytes, all participants underwent echocardiodoppler to assess left ventricular function, wall thickness and motion and valves’ functions; also, a Doppler of the renal arteries as well as urinary metanephrines and serum thyroid-stimulating hormone (TSH) were performed to rule out all causes of secondary hypertension. The mean ejection fraction recorded among participants was 68%, and none of them was found to have motion wall abnormalities, diastolic dysfunction nor significant valvulopathy. Renal arteries of all participants were patent with good Doppler signal and urinary metanephrine levels were normal. Selected subjects were randomly divided into four groups (three treatment and one placebo) each consisting of 7, 8 subjects based on their taste preference. Selected subjects were instructed to take a dose of 300 mL/day every morning before breakfast for 16 weeks. SBPs, DBPs and MAPs all participating subjects were monitored at weeks 4, 8, 12 and 16 by the same physician. Systolic and diastolic BPs were measured from the left arm using a mercury sphygmomanometer. Measurements were performed and repeated for three times at intervals of 10-min in a sitting position. Safety was assessed by general physical examination that was performed every two weeks, and subjects were regularly asked about experiencing any incidence of treatment-related adverse events throughout the treatment and post-treatment follow-up periods. Moreover, testing for liver and kidney functions was performed every 4th week (i.e., weeks 4, 8, 12 and 16) as well as four weeks after the end of the trail to assess for any post-treatment changes. All subjects showed normal values throughout the trial and during the follow-up period. 

### 2.4. Statistical Analysis

Statistical analysis was performed using the SPSS (IBM Corp. Released 2013, SPSS Statistics for Windows Version 22.0, Armonk, NY, USA). Categorical and continuous variables were expressed as frequencies and mean ± standard deviation, respectively. Quantitative variables were tested for normality distribution using the Kolmogorov–Smirnov test. Kruskal–Wallis test was used to study if there was a significant difference between the four groups (Placebo, *Mentha longifolia* (M.L.), *Viola odorata* (V.O.) and *Urtica dioica* (U.D.)) over the 16-week duration. Friedman test was used to show if there was a statistically significant difference in performance over time for the systolic (SBP) and diastolic blood pressures (DBP) as well as for the mean arterial blood pressures (MAP). Then a post hoc analysis with Wilcoxon signed-rank tests was used to confirm when the differences occurred compared to baseline. The level of significance was set at *p* < 0.05 for all statistical analyses. A priori statistical power analysis revealed that *n* = 29 was the suitable sample size in order to assess standardized differences in the main parameters at 0.05 significance level of two-sided hypothesis, reaching a power >95%.

## 3. Results

### 3.1. Ethnopharmacological Data

Table 1 illustrates a list of 26 native wild plant species cited by 36 herbalists and traditional healers as “widely used for the treatment of HTN in Lebanon”. The value of RFCs of most (19 out of 26) plants fell in the 0.72–0.95 range, with *M. longifolia*, *U. dioica* and *V. odorata* recording the highest values (0.95) followed by *A. ampeloprasum* (0.94), *A. graveolens* (0.92) and *C. azarolus* (0.90). According to informants, the perceived benefits and safety of cited species were the reasons for their popularity of use. All plant parts appeared to obtain some therapeutic benefits with leaves and aerial parts recording the highest citations (69.2%, 28.5% respectively). Notably, decoction was the principal means of preparation (65%) and oral administration at a dosage of 1–3 cups/day for a duration of 3–6 months (90%) was the main application method and the most efficacious dosage. 

### 3.2. Clinical Trial 

**Figure 1:**
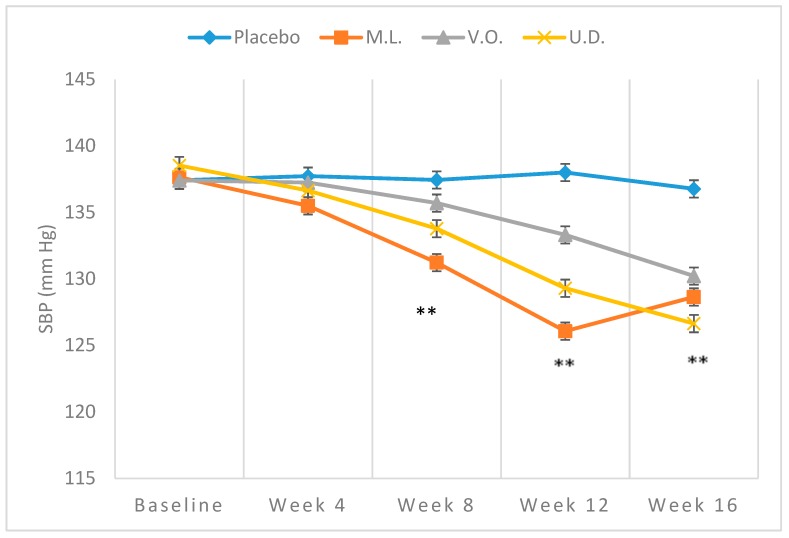
Means ± SD of SBP, measured over 16-week intake of 300 mL/day of *M. longifolia* (M.L.), *V.odorata* (V.O.) and *U. dioica* (U.D.) in mild hypertensive subjects. ** *p* < 0.01 (M.L. and U.D. compared to Placebo at week 8 and M.L., V.O., and U.D. compared to Placebo at weeks 12 and 16).

**Figure 2:**
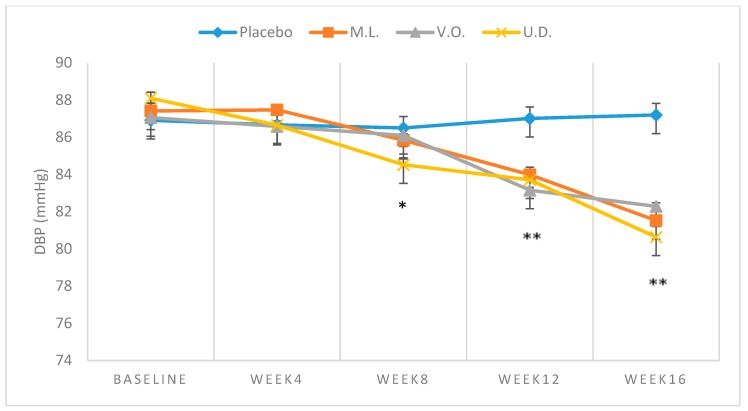
Means ± SD of DBP, measured over 16-week intake of 300 mL/day of *M. longifolia* (M.L.), *V.odorata* (V.O.) and *U. dioica* (U.D.) in mild hypertensive subjects.* *p* < 0.05 (U.D. compared to Placebo), ** *p* < 0.01 (M.L., V.O., and U.D. compared to Placebo).

**Figure 3:**
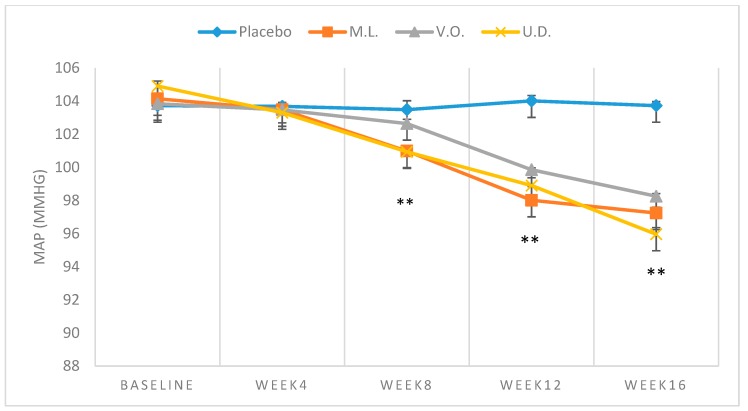
Means ± SD of MAP, measured over 16-week intake of 300 mL/day of *M. longifolia* (M.L.), *V.odorata* (V.O.) and *U. dioica* (U.D.) in mild hypertensive subjects. ** *p* < 0.01 (M.L. and U.D. compared to Placebo at week 8 and M.L., V.O., and U.D. compared to Placebo at weeks 12 and 16).

Demographic characteristics of the sampled population, all Caucasians, are shown in Table 2. Table 3 demonstrates the mean baseline levels of BP (*n* = 29). The subjects were men (19) and women (10) with an average age of 53.5 years. The mean values of baseline SBP, DBP or MAP of both plant-treated or the placebo groups fell in the range of 137.40 ± 1.50 to 138.44 ± 1.38 mmHg, 86.91 ± 1.63 to 87.80 ± 0.37 mmHg and 103.74 ± 1.19 to 104.68 ± 0.61 mmHg, respectively, with no significant difference (*p* > 0.01) between participating groups (Table 3). While no significant reduction (*p* > 0.01) was observed with BP of the placebo subjects over the 16-week trial, consistent reductions were clearly noted with the plant treated groups (Table 3). Comparison of the repeated measures for SBP using Friedman’s test showed a statistically significant difference over time of testing for M.L. (*χ*^2^ = 27.827, *p* < 0.001), V.O. (*χ*^2^ = 24.571, *p* < 0.001) and U.D. (*χ*^2^ = 31.119, *p* < 0.001). From baseline to week 16 of intake, SBP mean values fell from 137.64 ± 0.38 to 128.64 ± 0.38 mmHg (−9.00 ± 1.88 mmHg) (Figure 1), DBP from 87.41 ± 1.15 mmHg to 81.53 ± 1.49 mmHg (−5.89 ± 1.72) (Figure 2) and MAP from 104.14 ± 0.70 mmHg to 97.24 ± 0.95 mmHg (−6.92 ± 1.15) (Figure 3) with *M. longifolia*. Indeed, for M.L., Wilcoxon signed-rank tests showed that after week 4, there was a statistically significant decrease in the mean of SBP compared to baseline (*Z* = −2.530, *Z* = −2.530, *Z* = −2.530 or *Z* = −2.646, with *p* = 0.011, *p* = 0.011, *p* = 0.011 or *p* = 0.008, for weeks 4, 8, 12 or 16, respectively). Likewise, the values of SBP, DBP and MAP with *V. odorata* dropped from 137.40 ± 1.51 mmHg to 130.21 ± 0.79 mmHg (−7.19 ± 1.92), from 87.06 ± 2.04 mmHg to 82.29 ± 0.52 mmHg (−4.77 ± 1.83), and from 103.84 ± 1.65 mmHg to 98.26 ± 0.44 mmHg (−5.58 ± 1.45), respectively (Figure 1, Figure 2 and Figure 3). Wilcoxon signed-rank tests showed that after week 12, there was a statistically significant decrease in the mean of the SBP compared to baseline (*Z* = −2.456 or *Z* = −2.410, with *p* = 0.014 or *p* = 0.016, for weeks 12 or 16, respectively). As for *U. dioica*, more profound changes were observed with SBP dropping from 138.53 ± 1.31 mmHg to 126.64 ± 2.70 mmHg (−11.89 ± 2.60), DBP from 88.10 ± 0.91 mmHg to 80.64 ± 1.62 mmHg (−7.46 ± 1.16) and MAP from 104.91 ± 0.85 mmHg to 95.96 ± 1.13 mmHg (−8.94 ± 1.14) (Figure 1, Figure 2 and Figure 3). Wilcoxon signed-rank tests showed that after week 4, there was a statistically significant decrease in the mean of SBP compared to baseline (*Z* = −2.375, *Z* = −2.521, *Z* = −2.524 or *Z* = −2.533, with *p* = 0.018, *p* = 0.012, *p* = 0.011 and *p* = 0.012, for weeks 4, 8, 12 or 16 respectively).

For DBP, comparison of the repeated measures using Friedman’s test showed a statistically significant difference over time of testing for M.L. (*χ*^2^ = 25.928, *p* < 0.001), V.O. (*χ*^2^ = 22.377, *p* < 0.001) and U.D. (*χ*^2^ = 28.100, *p* < 0.001). For the M.L. group, Wilcoxon signed-rank tests showed that starting week 8, there was a statistically significant decrease in the mean of DBP compared to baseline (*Z* = −2.366, *Z* = −2.366 or *Z* = −2.384, with *p* = 0.018, *p* = 0.018 or *p* = 0.017, for weeks 8, 12 or 16, respectively). For V.O. group, it was observed that starting week 12, there was a statistically significant decrease compared to baseline (*Z* = −2.209 or *Z* = −2.375 with *p* = 0.027 or *p* = 0.018, for weeks 12 or 16, respectively). For the U.D. group, a significant difference was noted starting week 8 (*Z* = −2.521, *Z* = −2.524 or *Z* = −2.521, with *p* = 0.012, *p* = 0.012 or *p* = 0.012, for weeks 8, 12 or 16, respectively).

For MAP, comparison of the repeated measures using Friedman’s test showed a statistically significant difference over time of testing for M.L. (*χ*^2^ = 26.171, *p* < 0.001), V.O. (*χ*^2^ = 25.943, *p* < 0.001) and U.D. (*χ*^2^ = 31.300, *p* < 0.001). In particular, for the M.L. group, starting week 4, there was a statistically significant decrease in MAP compared to baseline (*Z* = −2.043, *Z* = −2.366, *Z* = −2.366 or *Z* = −2.388, with *p* = 0.041, *p* = 0.018, *p* = 0.018 or *p* = 0.017, for weeks 4, 8, 12 or 16, respectively). As for the V.O. group, a significant decrease compared to baseline was noted starting week 12 (*Z* = −2.371 or *Z* = −2.366, with *p* = 0.018 or *p* = 0.018, for weeks 12 or 16, respectively). In the U.D. group, a significant decrease compared to baseline was noted starting week 4 (*Z* = −2.521, *Z* = −2.524, *Z* = −2.521 or *Z* = −2.521, with *p* = 0.012, *p* = 0.012, *p* = 0.012 or *p* = 0.012, for weeks 4, 8, 12 or 16, respectively).

## 4. Discussion

The results of this ethnopharmacological survey indicate that 15 of the 26 reported species exhibit high RCF values, reflecting the significant consensus among informants about the presumed powerful therapeutic potential of these species [41]. Numerous previous ethnopharmacological studies have shown that all plants of the list are still being used for the treatment of HTN by many communities in different parts of the world [15,28,29,30,34,42,43,44,45,46,47]. In particular, the popular use of some species such as *A. ampeloprasum*, *A. graveolens*, *C. azarolus*, *M. longifolia*, *U. dioica* and *V. odorata* having very high RCF values (0.85–0.10) makes it tempting to speculate that their potential as a valuable source for pharmaceutical novel drug discovery is promising [48,49,50,51,52]. Pharmacological research and clinical trials have revealed the antihypertensive and vasodilatory activities of these species, further supporting this traditional use [53,54]. Food species such as *A. ampeloprasum*, *A. graveolens* and *C. azarolus*, among other species, appear to occupy considerable share of the list. This is mostly a result of the positive accumulated traditional experience derived from the consumption of such plants. 

The highly popular use of *C. azarolus* found in this study is also supported by the findings of other pharmacological studies and clinical trials [50,55]. Several mechanisms have been suggested for the observed hypotensive effects. These include a role for endothelium-dependent NO-mediated vasorelaxation and inhibition of Ca^2+^ influx to the smooth muscle [55]. Moreover, the role of antioxidant, anti-inflammatory and anti-proliferative activities was reported [56]. In addition, inhibition of angiotensin-converting enzyme by other Crataegus species has also been reported [57]. Such actions are credited to the plant’s multiple components such as flavonoids (hyperoside, quercetin, rutin, and vitexin), oligomeric proanthocyanidins [58] and quercetin [59]. Interestingly, some of these phytochemicals, such as isoflavones, exhibit estrogen-like effects [60]. Knowing that estrogen plays a very important role in hypertension [61], it would not be surprising that some of these phytoestrogens modulate blood pressure. Indeed, there is an inverse association between dietary intake of phytoestrogens and hypertension, both in the Mediterranean region [62] and elsewhere [63] as well as in animal models [64]. Indeed, we have recently discussed how flavonoids play an important role in the pathogenesis of hypertension [65].

The frequent use of *Artemisia herba-alba* Asso and *Peganum harmala* noted in this study is in full accordance with the results of a study from Morocco [43]. The hypotensive and vasodilatory effects of *P. harmala* (Syrian Rue or Esfand) have been associated with the activities of its isolated alkaloids [66,67]. Harmine, harmaline and harman were revealed to induce their actions by stimulating endothelial cells to release NO, blocking voltage-dependent Ca^2+^ channels, or inhibiting phosphodiesterases, thus resulting in an increase in cyclic AMP (cAMP) [68]. This cAMP not only stimulates relaxation of vascular smooth muscles cells [68] but also modulates trafficking of α2C-adrenoceptors to cell surface, thus making it readily available for epinephrine, its natural agonist [18,19,69]. 

The use of the plants we listed above as a mode of alternative or complementary medicine is believed to be largely attributed to the deep-rooted belief in the healing potential, accessibility and lower risk of plants compared with synthetic drugs. Despite the promising therapeutic potential of the cited species, informants were fully aware of the possible consequences of overuse and drug interactions, particularly in the case *P. harmala* and *A. herba alba*. This belief is supported by convincing arguments regarding the safety of both species as they have been reported to cause side effects in animal and human case reports [70,71,72]. Importantly, some of the side effects may also be due to herb–drug interactions, especially, that the concurrent use of both traditional and pharmaceutical medications for treating hypertension or other chronic diseases is a worldwide tendency [73]. Indeed, a study among hypertensive patients in Palestine revealed a majority of patients using herbal medicines did not disclose this fact to their health providers [74]. 

Clear evidence of adverse reactions has indeed been reported [73,75]. Intriguingly, edible plants such as *A. ampeloprasum*, *A. graveolens* and *C. azarolus*, *M. longifolia*, *U. dioica* and *V. odorata* among others that are well established for their potential in alleviating hypertension may be considered as nontoxic under moderate use. In this context, it is noteworthy that the importance placed by informants on *M. longifolia*, *U. dioica* and *V. odorata* was remarkable. It was therefore believed that additional insights into the use of these species may be gained by conducting a clinical trial that may contribute to the development of effective, safe and perhaps novel moieties or formulations to curb the prevalence of HTN among Lebanese people. 

The results indicated in Table 3 clearly indicate significant drops in SBP, DBP and MAP that are duration-dependent. The reductions were particularly significant at weeks 12 and 16 with three plants indicating the benefits of the tested plants in complementary therapy for mild hypertensive subjects and supporting their traditional use. A reduction of 4–5 mmHg in SBP and 2–3 mmHg in DBP has been estimated to reduce the risk of cardiovascular morbidity and mortality by 8–20% [76]. However, the high SD values in some values reflect wide divergence from the means and high fluctuations, thus highlighting the need for more trials. The examination of the effectiveness of extract in forestalling the progression of mild hypertension into a hypertensive state, as observed with certain antihypertensive pharmaceutical medications, is also necessary [77]. 

Despite accumulated data, there are currently no clinical studies on the purported effectiveness of the tested plants. Thus, our findings can only be discussed in view of ethnopharmacological studies and wide spectrum of pharmacological activities involving tissue preparations and in vivo animal models. Specifically, the aqueous methanolic extract of *M. longifolia* was revealed to have significant antihypertensive and negative chronotropic in normotensive and induced hypertensive rats [54]. This hypotensive effect was further confirmed by a more recent study of the crude extract of leaves and its chloroform and aqueous fractions producing a dose-dependent fall in MAP in normotensive anesthetized rats [78]. The functional nature of the blood-pressure-lowering effect was further studied using isolated aortic ring rat preparations of rabbits, rats and guinea pigs [78]. The crude extract was found to possess a combination of vasodilator and cardiac depressant constituents responsible for the blood pressure lowering effect. The vasodilatory effect was mediated through a combination of Ca^+2^ channel blockade (concentrated in a non-polar fraction) and endothelium-dependent pathway linked to vascular muscarinic receptors (concentrated in a polar fraction) [78]. The role of antioxidant effect of phenolics and total flavonoids contents was also reported [79,80]. 

In this study, only a slight decrease in SBP at a dose of 45 mL/day of squeezed leaf juice for two weeks was recorded. It may be argued that higher doses or longer durations may be required to induce more effective BP-lowering effect evidently indicated in Table 2 and Figure 1. This is in agreement with the results of the examination of the ethanolic extract of *U. dioica* leaves, which was found to significantly decrease elevated BP in renal artery-occluded hypertensive rats in a dose-dependent manner [81]. More recently, the crude methanolic extract of the dried rhizomes and its fractions were shown to significantly reduce blood pressure in high NaCl induced hypertensive rats under anesthesia [82]. The in vitro examination on rat and rabbit aorta rings attributed this effect to NO mediated vasorelaxation and Ca^+2^ blocking effects involving both endothelial cells and smooth muscle fibers. *Urtica dioica* supplementation was also found to increase plasma antioxidant capacity and reduce systemic oxidative stress [51]. The finding of the significant drop in both SBP, DBP and MAP herein indicated together with the results of previous pharmacological studies are consistent with the reported high content of *U. dioica* of bioactive phenolic compounds and other compounds known to have significant antioxidant activity and vasorelaxant properties with various proposed underlying mechanisms of action [83,84]. 

The hypotensive effect of *V. odorata* in this study concurs with the results of the dose-dependent lowering effect of MAP found in anaesthetized rats [52]. In isolated guinea-pig atria, the extract showed negative inotropic and chronotropic effects, similar to that caused by verapamil, a standard Ca^+2^ antagonist known to cause cardiac depression through the inhibition of Ca^+2^ inward current during the action potential plateau [52]. This indicated that the observed cardiac inhibitory effect of the plant extract might be causing a decrease in cardiac output and ultimately a decrease in the blood pressure. When tested in pre-contracted rat aortic preparations, the plant’s extract inhibited both high K^+^ and phenylephrine (PE) induced vasoconstriction by blockage of Ca^2+^ influx through voltage-dependent channels and receptor-operated channels caused by high K^+^ and PE, respectively [85]. In addition, when the control responses of PE were taken in Ca^2+^ free medium, the crude extract inhibited the PE-induced peaks, indicating that the inhibition of Ca^2+^ release from internal stores through inositol-1,4, 5-trisphosphate-sensitive sarcoplasmic reticulum mechanism [86,87]. 

The antioxidant potency of *V. odorata* was confirmed in other studies [88,89]. Furthermore, blocking voltage-dependent Ca^2+^ channels or suppressing Ca^2+^ release from the sarcoplasmic reticulum in PE-induced or spontaneously contracting isolated rabbit tissue preparations was also confirmed in a recent study [90]. Importantly, a phytochemical screening of *V. odorata* extracts and essential oils revealed the presence of a wide range of bioactive compounds [90,91], making it an attractive plant for further cardiovascular investigations. 

## 5. Conclusions

This study presents the first scientific evidence regarding the antihypertensive effects of *M. longifolia*, *V. odorata* and *U. dioica*, three commonly used plants of the Lebanese flora. The perceived benefits and safety of the discussed species were the reasons for their popularity of use. The clinical trial we conducted further supports the antihypertensive potential of these plants, especially that the extracts were well-tolerated without any clinically significant effects. For this reason, extracts of these plants present an effective, safe and promising potential as a phyto-therapeutical approach in the treatment and management of mild hypertension. Nonetheless, one major limitation of this study is the absence of a dose–response that could be used to better assess the pharmacological responses. Another limitation is the lack of accurate characterization, such as by mass spectrometry or high-performance liquid chromatography (HPLC), of the extracts. Therefore, more research on the pharmacological effects and the underlying mechanisms is still warranted. 

## Figures and Tables

**Table 1:** Plant species and traditional practices traditionally used for the treatment hypertension (HTN) in Lebanon.

Plant Species (Family)	English Name	Arabic Name	Preparation and Administration	RFCs
*Allium ampeloprasum* L. (Amaryllidaceae)	Leek	Kerrat	Decoction of bulbs and leaves, 1 cup/day. Medicinal food	0.94
*Apium graveolens* L. (Apiaceae)	Wild Celery	Krafs	Fresh juice of shoots and leaves, 1 cup twice/week	0.92
*Artemisia herba alba* Asso (Asteraceae)	White Worm-wood	Shieh	Infusion of aerial parts, 1 cup/day	0.64
*Asparagus acutifolius* L. (Asparagaceae)	Wild Asparagus	Halyoun	Decoction of stem tops, 1 cup/day	0.90
*Calicotome villosa* (Poir.) Link (Fabaceae)	Spiny broom	Kandoul	Decoction of seeds, 1 cup/day	0.35
*Centaurium erythraea* Rafn (Gentianaceae)	Spiked centaury	Kantarioun	Infusion of flowering aerial parts, 3 cups/day for 2 weeks	0.55
*Crataegus azarolus* L. (Rosaceae)	Hawthorn	Zaarour	Decoction of leaves, flowers or fruits 1 cup/day	0.90
*Cupressus sempervirens* L. (Cupressaceae)	Cypress	Sarou	Decoction of leaves, 1 cup/day	0.45
*Equisetum telmateia* Ehrh. (Equisetaceae)	Branched horsetail	Zanab El-khayl	Aerial parts Infusion/3cups/day for 8–12 weeks	0.75
*Eryngium creticum* Lam. (Apiaceae)	Eryngo	Kers Aanni	Juice of young shoots and leaves, ½ cup/day	0.80
*Foeniculum vulgare* Mill	Fennel	Choumar	Decoction of seeds, 2 cups/day	0.65
*Fibigia clypeata* (L.) Medik. (Brassicaceae)	Roman Shields	Hachichet El Oumeh	Infusion of leaves, 1cup/day	0.90
*Hordeum vulgare* L. (Poaceae)	Barley	Sha’ir	Decoction of seeds, 1 cup/day	0.94
*Laurus nobilis* L. (Lauraceae)	Sweet bay	Ghar	Decoction of leaves, 1/2 cup/day	0.89
*Matricaria aurea* (Loefl.) Sch.Bip. (Compositae)	Chamomile	Bebounej	Infusion flowers, 3 cup/day as herbal tea	0.85
*Matricaria chamomilla* L. (Asteraceae)	Chamomile	Bebounej	Infusion of flowers, 3cup/day	0.85
*Mentha longifolia* L. (Lamiaceae)	Horse Mint	Na’na’a	Infusion of leaves, 2cup/day	0.95
*Melissa officinalis* L. (Lamiaceae)	Lemon Balm	Mallieseh	Infusion of leaves, 2cup/day	0.45
*Myrtus communis* L. (Myrtaceae)	Myrtle	Hemblas	Maceration of fresh fruits in oil, essential oil	0.86
*Paronychia argentea* Lam. (Caryophyllaceae)	Silvery Paronychia	Hachichet El Ramel	Decoction of aerial parts, 1 cup/day	0.40
*Peganum harmala* L. (Nitrariaceae)	Syrian rue, harmel	Harmala	Decoction of aerial parts, 1 cup/day	0.72
*Plantago major* L. (Plantaginaceae)	Broadleaf plantain	Lissan el Hamal	Decoction, 1 cup/day	0.89
*Portulaca oleracea* L. (Portulacaceae)	Purslane	Bakleh	Decoction of leaves, 3 cups/day	0.88
*Raphanus raphanistrum* L. (Brassicaceae)	Wild radish	Fejel Barie	Juice of aerial parts, roots Fresh 1/2 cup/day	0.94
*Urtica dioica* L. (Urticaceae)	Stinging nettle	Korrays	Decoction of young shoots and leaves, 3 cups/day	0.95
*Viola odorata* L. (Violaceae)	Sweet violet	Banafsaj	Infusion of flowers., 3 cup/day	0.95

RFC: relative frequency of citation.

**Table 2:** Demographic characteristics of the sampled population.

Characteristics	Treated Group (*n* = 22)	Placebo (*n* = 7)
**Age Groups (years)**		
40–47	7	2
48–57	9	3
58–65	6	2
**Gender**		
Men	15	4
Women	7	3
**Risk Factors**		
Smoking	22	7
Family history	22	7
**Body Mass Index (Mean)**		
Overweight (20–25)	16	2
Obese (>30)	6	5

**Table 3:** Means ± SD of SBP, DBP and MAP measured over 16 week intake of 300 mL/day of *M. longifolia* (M.L.), *V.odorata* (V.O.) and *U. dioica* (U.D.) in mild hypertensive subjects.

Group	SBP Mean ± SD				DBP Mean ± SD				MAP Mean ± SD			
	M.L. (*n* = 7)	V.O. (*n* = 7)	U.D. (*n* = 8)	Placebo (*n* = 7)	M.L. (*n* = 7)	V.O. (*n* = 7)	U.D. (*n* = 8)	Placebo (*n* = 7)	M.L. (*n* = 7)	V.O. (*n* = 7)	U.D. (*n* = 8)	Placebo (*n* = 7)
Baseline	137.64 ± 0.38	137.40 ± 1.51	138.53 ± 1.31	137.41 ± 0.89	87.41± 1.15	87.06 ± 2.04	88.10 ± 0.91	86.91 ± 1.64	104.14 ± 0.70	103.84 ± 1.65	104.91 ± 0.85	103.73 ± 1.19
Week 4	135.50 ^a^ ± 0.00	137.24 ± 0.24	136.64 ^a^ ± 1.40	137.73 ± 0.08	87.47 ± 0.79	86.59 ± 0.76	86.65 ± 0.47	86.67 ± 0.98	103.49 ^a^ ± 0.52	103.47 ± 0.48	103.30 ^a^ ± 0.62	103.69 ± 0.63
Week 8	131.23 ^a^ ± 1.13	135.70 ± 0.38	133.78 ^a^ ±1.59	137.44 ± 1.22	85.84 ^a^ ± 0.89	86.10 ± 1.00	84.53 ^a^ ± 1.03	86.50 ± 2.00	100.99 ^a^ ± 0.72	102.64 ± 0.68	100.94 ^a^ ± 1.01	103.49 ± 1.41
Week 12	126.07 ^a^ ± 1.51	133.31 ^a^ ± 0.38	129.30 ^a^ ± 0.69	138.00 ± 1.00	83.99 ^a^ ± 0.71	83.16 ^a^ ± 0.38	83.71^a^ ± 1.92	87.01± 1.25	98.01 ^a^ ± 0.58	99.86 ^a^ ± 0.31	98.90 ^a^ ± 1.35	104.01 ± 0.85
Week 16	128.64 ^a^ ± 0.38	130.21 ^a^ ± 0.79	126.64 ^a^ ± 2.70	136.77 ± 1.06	81.53 ^a^ ± 1.49	82.29 ^a^ ± 0.52	80.64 ^a^ ± 1.62	87.20 ± 0.77	97.24 ^a^ ± 0.95	98.26 ^a^ ± 0.44	95.96 ^a^ ± 1.13	103.73 ± 0.66

SD: standard deviation; SBP: systolic blood pressure; DBP: diastolic blood pressure; MAP: mean arterial blood pressure; ^a^ represents values that are significantly different at *p* < 0.01 arterial blood pressure.
